# RORγt Inhibitor-SR1001 Halts Retinal Inflammation, Capillary Degeneration, and the Progression of Diabetic Retinopathy

**DOI:** 10.3390/ijms21103547

**Published:** 2020-05-17

**Authors:** Thomas E. Zapadka, Sarah I. Lindstrom, Brooklyn E. Taylor, Chieh A. Lee, Jie Tang, Zakary R. R. Taylor, Scott J. Howell, Patricia R. Taylor

**Affiliations:** 1 Department of Ophthalmology and Visual Sciences, Case Western Reserve University, School of Medicine, Cleveland, OH 44106, USA; tez9@case.edu (T.E.Z.); sxl926@case.edu (S.I.L.); taylo465@miamioh.edu (B.E.T.); chiehallen_lee@yahoo.com (C.A.L.); jxt50@case.edu (J.T.); zxt154@case.edu (Z.R.R.T.); showell144@gmail.com (S.J.H.); 2 Louis Stokes Cleveland VA Medical Center, Cleveland, OH 44106, USA

**Keywords:** diabetic retinopathy, RORγt, SR1001, retinal inflammation, capillary degeneration

## Abstract

Diabetic retinopathy is a diabetes-mediated retinal microvascular disease that is the leading cause of blindness in the working-age population worldwide. Interleukin (IL)-17A is an inflammatory cytokine that has been previously shown to play a pivotal role in the promotion and progression of diabetic retinopathy. Retinoic acid-related orphan receptor gammaT (RORγt) is a ligand-dependent transcription factor that mediates IL-17A production. However, the role of RORγt in diabetes-mediated retinal inflammation and capillary degeneration, as well as its potential therapeutic attributes for diabetic retinopathy has not yet been determined. In the current study, we examined retinal inflammation and vascular pathology in streptozotocin-induced diabetic mice. We found RORγt expressing cells in the retinal vasculature of diabetic mice. Further, diabetes-mediated retinal inflammation, oxidative stress, and retinal endothelial cell death were all significantly lower in RORγt^−/−^ mice. Finally, when a RORγt small molecule inhibitor (SR1001) was subcutaneously injected into diabetic mice, retinal inflammation and capillary degeneration were ameliorated. These findings establish a pathologic role for RORγt in the onset of diabetic retinopathy and identify a potentially novel therapeutic for this blinding disease.

## 1. Introduction

Diabetes mellitus affects millions of individuals across the globe and its prevalence is on the rise. Recent predictions estimate that by 2045 nearly 630 million people will be diagnosed with diabetes [1]. As diabetes progresses, more than 60% of Type II and 95% of Type I diabetics develop diabetic retinopathy. Making diabetic retinopathy the leading cause of blindness in the working-age population worldwide [2]. Diabetes-mediated hyperglycemia induces chronic, low-grade inflammation that elicits gradual, asymptomatic alterations in the retinal microvasculature. Inflammation initiates blood–retina-barrier (BRB) permeability, immune cell leukostasis, and oxidative stress [3,4,5,6]. This causes capillary non-perfusion, which is one of the earliest detectable signs of clinical non-proliferative diabetic retinopathy [7,8]. In response to this vasoregression, angiogenesis of retina vessels is induced, leading to proliferative diabetic retinopathy and vision loss [9,10,11].

Diabetes induces Interleukin (IL)-17A production, which is one of the most prevalent cytokines associated with inflammatory pathogenesis [12]. In Type I diabetes, an autoimmune dysfunction initiates the differentiation of Th17 cells that destroy β cells in the pancreatic islets, impairing insulin secretion and glucose metabolism [13,14]. In Type II diabetes, a dysfunction in adipocytes initiates IL-6 and excess leptin production, which promotes the differentiation of Th17 cells [15,16,17]. Persistence of circulating Th17 cells and IL-17A in both Type I and Type II diabetics has been correlated to the onset and progression of diabetic complications [18,19,20,21,22]. Further, in diabetic mice it was determined that IL-17A played a pivotal role in BRB permeability, vascular impairment, and the onset of non-proliferative diabetic retinopathy [23,24,25,26]. 

Retinoic acid-related orphan receptor gammaT (RORγt) is a ligand-dependent transcription factor that mediates IL-17A production [12,27]. When activated, RORγt translocates to the nucleus and binds to the *Il17* gene, which up-regulates transcription and production of IL-17A [28]. In diabetes, a combination of inflammation and hyperglycemia activates RORγt [27]. Although the role of RORγt in the onset of diabetic retinopathy is not yet known, there is evidence that links RORγt to the progression of other diabetic complications and retinal neovascularization in oxygen induced retinopathy [29,30,31,32]. Taken together, we postulated that RORγt plays a pivotal role in the pathogenesis of non-proliferative diabetic retinopathy. Further, it was our goal to identify a potential therapeutic that would delay the onset of diabetic retinopathy and inhibit vision loss.

In the current study, RORγt expressing cells were detected in the sera and retinal vasculature of streptozotocin (STZ)-induced diabetic mice. Ablation of RORγt in RORγt^−/−^ diabetic mice significantly decreased retinal inflammation, oxidative stress, and retinal endothelial cell death. These observations were extended by therapeutically administering a RORγt small molecule inhibitor-SR1001 to diabetic mice, wherein blocking RORγt activity impaired retinal capillary degeneration. These are the first findings to establish a pathologic role for RORγt in diabetes-mediated retinal capillary non-perfusion, as well as identify a potentially novel therapeutic for the onset and progression of diabetic retinopathy. 

## 2. Results

### 2.1. Hyperglycemia in STZ-Induced Diabetic Mice

**Figure 1:**
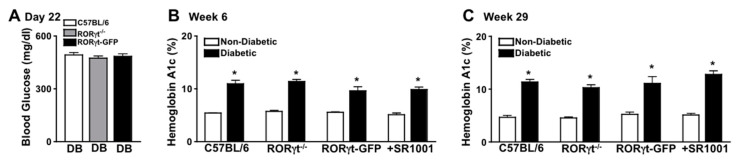
Hyperglycemia in streptozotocin (STZ)-induced diabetic C57BL/6 and Retinoic acid-related orphan receptor gammaT (RORγt) transgenic mice. (**A**) Assessment of 6-h fasted Blood Glucose in diabetic C57BL/6 (white), RORγt^−/−^ (grey), and RORγt-GFP (black) mice (*n* = 20/group), 17 days after STZ injections (Day 22). Glycated Hemoglobin A (A1c) in non-diabetic (white) and STZ-induced diabetic (black) mice at 6 weeks (**A**) and at 29 weeks (**C**) after diabetic conditions were confirmed in C57BL/6, RORγt^−/−^, RORγt-GFP, and SR1001 treated mice. Error bars represent the standard error of the mean (SEM), and * *p* < 0.01. Data are representative of three separate experiments.

Diabetes-mediated hyperglycemia was sustained throughout a 2-month (*n* = 20/group) or an 8-month (*n* = 7/group) period in STZ-induced diabetic mice. Fasted (6 h) blood glucose levels were measured 17 days after the last STZ-injection to confirm diabetic conditions, whereas all diabetic groups had an average blood glucose level of ~480 mg/dl (Figure 1A). Non-fasted blood glucose levels were also quantified at week 6 and 29, wherein glucose levels were >600 mg/dl (data not shown). Further, sera were evaluated in non-diabetic and STZ-diabetic mice to quantify A1c levels of hyperglycemia at week 6 and 29. The severity of hyperglycemia was similar (with no significant differences) among diabetic wild type (C57BL/6), RORγt-GFP, and RORγt^−/−^ mice, as well as SR1001 treated diabetic C57BL/6 mice (Figure 1B,C). 

### 2.2. RORγt Expressing Cells in the Retinal Vasculature of Diabetic Mice

**Figure 2:**
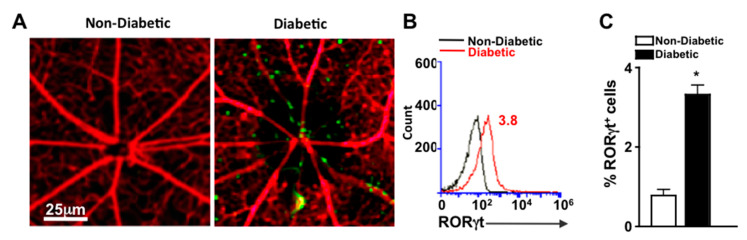
RORγt-GFP^+^ cells in the retinal vasculature (**A**) Representative fluorescent microscopy images of RORγt/GFP^+^ cells in retinas of non-diabetic and diabetic RORγt-GFP reporter mice (*n* = 3/group) following perfusion and vascular staining (scale bars of all images = 25 μm, which is a visual indicator of the size of the representative image). (**B**) Representative flow cytometry overlay of RORγt/GFP^+^ cells in the retina vasculature of non-diabetic (black) and STZ-induced diabetic (red) RORγt-GFP mice. (**C**) Flow cytometry quantification (*n* = 5 pooled samples (15 mice)/group) of percent positive (of 10,000 events) RORγt/GFP^+^ cells in retinas of non-diabetic (white) and diabetic (black) RORγt-GFP mice. Error bars represent the SEM, and * *p* < 0.01 per unpaired student’s t-test. All data was collected 2 months after diabetes was confirmed. Data are representative of two separate experiments with similar results.

To detect cells that express RORγt in the retinal vasculature, we examined retinas of reporter mice that express functional RORγt reported by GFP expression (RORγt-GFP mice). Vessels were perfused, stained red with Rhodamine, and retina whole mounts were examined microscopically for the presence of RORγt-GFP cells. As shown in representative images, RORγt/GFP^+^ cells were adhered to the retinal vasculature of diabetic, but not non-diabetic mice (Figure 2A). To quantify the cells, retinas were digested, and cells of the retina and retinal vasculature were analyzed by flow cytometry analysis. No RORγt/GFP^+^ cells were detected in the retinas of non-diabetic mice; however, 3.8% of total cells in the retina and retinal vasculature of diabetic mice were RORγt/GFP^+^ (Figure 2B). Similar results identifying RORγt expressing cells in diabetic retinas were observed in five separate samples (Figure 2C).

### 2.3. Systemic Ablation of RORγt Decreases Retinal Oxidative Stress and Inflammation

**Figure 3:**
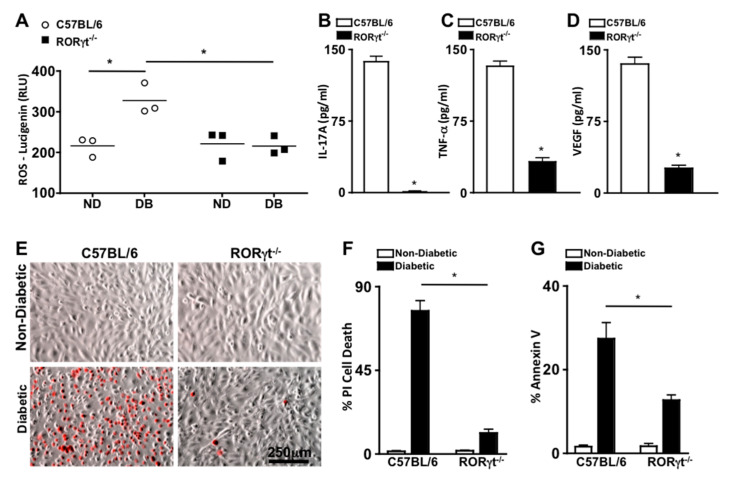
Oxidative stress, retinal inflammation, and endothelial cell death in retinas. (**A**) Quantification of reactive oxygen species (ROS) in the retinas of non-diabetic (ND) and diabetic (DB) C57BL/6 (white circles) and RORγt^−/−^ (black squares) mice; each data point represents an individual retina (*n* = 3). ELISA quantifications of IL-17A (**B**), TNF-α (**C**), and VEGF (**D**) in retinas (*n* = 3) of diabetic C57BL/6 (white) and RORγt^−/−^ (black) mice. ROS and inflammatory protein analysis were performed 2 months after diabetic conditions were confirmed. (**E**) Representative images of murine retinal endothelial cells (mREC) co-cultured with T cells of non-diabetic (top) and diabetic (bottom) C57BL/6 or RORγt^−/−^ mice 48 h after incubation; dead red cells are Propidium iodide (PI) positive (scale bars of all images = 250 μm, which is a visual indicator of the size of the representative images). Flow cytometry quantifications of percent CD144^+^/PI^+^ cell death (**F**) or Annexin V^+^ apoptosis (**G**) in mREC co-cultured (*n* = 6) with T cells of non-diabetic (white) or diabetic (black) C57BL/6 or RORγt^−/−^ mice. Percent positive cells are from analysis of 30,000 events. Error bars represent the SEM, and * *p* < 0.001 per unpaired student’s *t*-test.

To ascertain the role of RORγt in retinal oxidative stress during diabetes, reactive oxygen species (ROS) was quantified 2 months after diabetic conditions were confirmed in C57BL/6 and RORγt^−/−^ mice (*n* = 3). ROS was significantly increased in the retinas of diabetic compared with non-diabetic mice, which was significantly lowered in the diabetic RORγt^−/−^ mice (Figure 3A). Additionally, the levels of ROS in the retinas of diabetic RORγt^−/−^ mice were similar to that of all non-diabetic mice (Figure 3A). 

To evaluate the role of RORγt in diabetes induced retinal inflammation, inflammatory proteins previously identified as precursors to the onset of diabetic retinopathy (IL-17A, TNF-α (Tumor Necrosis Factor-alpha), and VEGF (Vascular Endothelial Growth Factor)) were examined [5,21]. Retinal protein lysates from diabetic C57BL/6 and RORγt^−/−^ mice (*n* = 3) were collected 2 months after diabetic conditions were confirmed, and levels of IL-17A, TNF-α, and VEGF were quantified by ELISA analysis. As shown in Figure 3B–D, ~140 pg/mL of IL-17A, ~125 pg/mL of TNF-α, and ~135 pg/mL of VEGF were detected in the retinas of diabetic wild type (C57BL/6) mice. Conversely, all inflammatory proteins were significantly decreased in the retinas of diabetic RORγt^−/−^ mice, whereas only negligible levels of IL-17A (Figure 3B), ~25 pg/mL of TNF-α (Figure 3C), and ~20 pg/mL of VEGF (Figure 3D) were detected. Similar results were detected in a separate replicated experiment. Taken together, these results signify a role for RORγt in diabetes-mediated retinal inflammation and oxidative stress.

### 2.4. RORγt Enhances Retinal Endothelial Cell Death

Previously, we determined that Th17 cells adhere to the retinal vasculature, wherein they produce IL-17A that can bind to retinal endothelial cells. When IL-17A binds to its IL17R receptor, an Act1-FADD signaling cascade induces retinal endothelial cell death, vascular impairment, and the onset of non-proliferative diabetic retinopathy in diabetic mice [25,26]. In the current study, we detected RORγt expressing cells migrating through and adhered to the retinal vasculature of diabetic mice (Figure 2A). Since T cells are the most prevalent RORγt expressing cell [12,27], we wanted to further determine the role of RORγt^+^/T cells in retinal endothelial cell death. Hence, we co-cultured murine retinal endothelial cells (mREC) with T cells from non-diabetic and diabetic C57BL/6 or RORγt^−/−^ mice, stained the cells with Propidium iodide ((PI), which is a red indicator for membrane permeability seen in dead cells), and examined mREC cell viability by live cell images (Figure 3E) and flow cytometry analysis (Figure 3F). After 48 h, there was a significant increase in retinal endothelial cell death (CD144^+^/PI^+^) in mREC co-cultured with diabetic than non-diabetic T cells (Figure 3F). Yet, as shown in Figure 3F, there was a significant decrease in retinal endothelial cell death in mREC co-cultured with T cells of diabetic RORγt^−/−^ mice (~11%) than diabetic-C57BL/6 mice (~76%). Taken together, this indicates that RORγt^+^/Tcells play a role in retinal endothelial cell death, which is the hallmark for capillary non-perfusion.

We also analyzed early stage apoptosis in co-cultured mREC with T cells from non-diabetic and diabetic C57BL/6 or RORγt^−/−^ mice through Annexin V analysis. We found that ~27% of mREC co-cultured with T cells of diabetic C57BL/6 mice were Annexin V positive compared to ~12% of mREC co-cultured with T cells of diabetic RORγt^−/−^ mice (Figure 3G). Collectively, this indicates that RORγt plays a pivotal role in diabetes-mediated apoptosis and programmed retinal endothelial cell death, which are precursors to retinal capillary non-perfusion and the onset of non-proliferative diabetic retinopathy.

### 2.5. RORγt Small Molecule Inhibitor—SR1001 Decreases Retinal Inflammation

**Figure 4:**
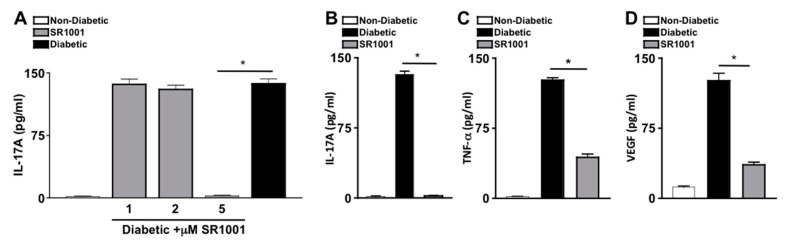
Inflammation in diabetic mice injected with RORγt inhibitor—SR1001 (**A**) Dose-dependent analysis of the efficacy of SR1001 to inhibit IL-17A production in sera 2 months after diabetic conditions were confirmed (*n* = 3), wherein IL-17A in the sera of non-diabetic (white), diabetic (black), or diabetic C57BL/6 mice treated with 1, 2, or 5 μM of RORγt inhibitor-SR1001 (grey) was quantified by ELISA. ELISA quantifications of IL-17A (**B**), TNF-α (**C**), and VEGF (**D**) in retinas (*n* = 3) of non-diabetic (white), diabetic (black), or diabetic treated with 5μM of SR1001 (grey) C57BL/6 mice. Error bars represent the SEM, and * *p* < 0.001, wherein *p*-value was first equated by two-way ANOVA analysis and then an unpaired t-test with Tukey’s post-hoc analysis. Data are representative of 2 separate experiments with similar results.

SR1001 is a small molecule inverse agonist of RORγt, which represses transcriptional activity at the *Il17* promoter and inhibits IL-17A production [33,34]. To ascertain the efficacy of SR1001 on diabetes induced IL-17A, sera of untreated non-diabetic and untreated or SR1001 treated diabetic mice were analyzed by ELISA. Diabetic C57BL/6 mice received 100 μL injections of sterile saline containing 1, 2, or 5 μM of SR1001. Mice received one injection per week for two months. Two months after diabetes was confirmed, sera were collected for IL-17A ELISA analysis. There were no differences in body weight, lethargy, mortality rate, respiratory stress, or organ appearance in the SR1001 treated mice compared to the untreated mice. As shown in Figure 4A, there was only negligible levels of IL-17A detected in the sera of non-diabetic mice, which was significantly increased to ~140 pg/mL in the sera (*n* = 3) of untreated diabetic mice, and diabetic mice that received 1 or 2 μM of SR1001 injections. However, IL-17A was significantly decreased to negligible levels in the sera of diabetic mice that received 5 μM SR1001 injections (Figure 4A). Since 5 μM injections of SR1001 were sufficient to ablate IL-17A in the sera of diabetic mice, and no toxic effects were observed, this treatment regimen was used throughout the remaining SR1001 treatment studies. 

To further evaluate the efficacy of the RORγt inihibitor-SR1001 in diabetes induced retinal inflammation, levels of IL-17A, TNF-α, and VEGF in the retina were quantified by ELISA. Retina protein lysates from untreated non-diabetic and diabetic C57BL/6 mice, as well as diabetic mice receiving weekly 5 μM injections of SR1001 were analyzed by ELISA, 2 months after diabetic conditions were confirmed (*n* = 3). As shown in Figure 4B–D, ~130 pg/mL of IL-17A, ~110 pg/mL of TNF-α, and ~125 pg/mL of VEGF were detected in the retinas of diabetic C57BL/6 mice. While only negligible levels of IL-17A (Figure 4B) and TNF-α (Figure 4C), and ~15 pg/mL of VEGF (Figure 4D) were detected in the retinas of non-diabetic mice. Yet, all inflammatory proteins were significantly decreased in the retinas of diabetic mice receiving subcutaneous SR1001 injections, with only negligible levels of IL-17A detected. Taken together, these results identify a novel therapeutic that can inhibit retinal inflammation and potentially delay the onset of diabetic retinopathy.

### 2.6. RORγt Small Molecule Inhibitor—SR1001 Significantly Decreases Leukostasis in Retinas of Diabetic Mice

**Figure 5:**
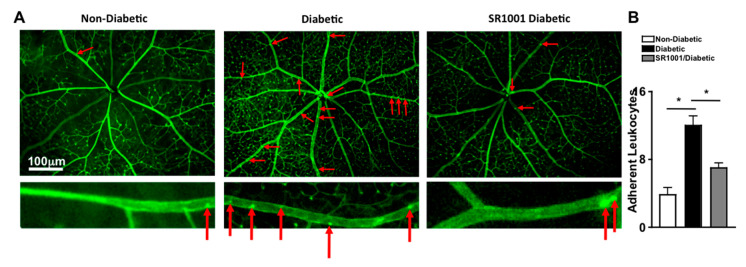
Leukostasis in the retinal vasculature of SR1001 treated diabetic mice. (**A**) Representative fluorescent microscopy images (scale bars of all images = 100 μm, which is a visual indicator of the size of the images) of leukocytes adhered to the retinal vasculature (highlighted by red arrows) in non-diabetic, diabetic, and SR1001 treated diabetic C57BL/6 mice following perfusion and vascular staining. Lower images are an individual retina vessel magnified 40× with adherent leukocytes highlighted by red arrows. (**B**) Quantification of adherent leukocytes to the vasculature in the retinas (*n* = 3) of 3 mice/group (**B**). Error bars represent the SEM, and * *p* < 0.01, wherein *p*-value was first equated by two-way ANOVA analysis and then an unpaired t-test with Tukey’s post-hoc analysis.

Previously, it was determined that diabetes mediates leukostasis, which is the adhesion of leukocytes to the capillary endothelium and the retinal vessel walls. Leukostasis can lead to capillary non-perfusion and vascular impairment in the retina [4]. To ascertain the efficacy of SR1001 in this early stage vascular pathology in the retina, vessels were perfused, adherent cells and retinal vasculature was stained green with FITC, flat mounts of retinas were imaged by fluorescent microscopy, and the number of adherent leukocytes were manually quantified. As shown in representative images (red arrows highlight the adherent leukocytes), there were very few leukocytes adhered to the retinal vasculature in non-diabetic mice, but a significantly higher number of adherent leukocytes were detected in the retinal vasculature in diabetic mice (Figure 5A). However, leukostasis was significantly decreased in the retinas (*n* = 3) of diabetic mice that received SR1001 injections compared to untreated diabetic mice (Figure 5B). These results suggest that SR1001 is sufficient to significantly decrease diabetes-mediated leukostasis and the onset of capillary non-perfusion.

### 2.7. SR1001 Ablates Capillary Degeneration in Diabetic Retinas

**Figure 6:**
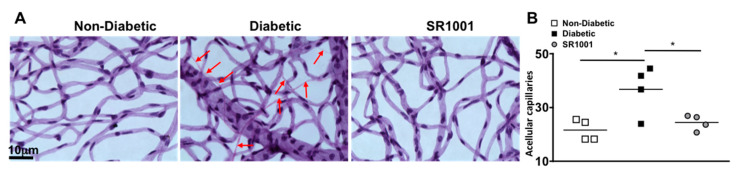
Acellular capillaries in the retinas of STZ-induced diabetic mice (**A**) Representative images of acellular capillaries and degeneration in retinal capillary beds of non-diabetic, diabetic, and SR1001 treated diabetic C57BL/6 mice (scale bars of all images = 10 μm, which is a visual indicator of the size of the representative images). Red arrows highlight acellular capillaries. (**B**) Quantification of acellular capillaries within a 1.10 mm^2^ area of each retina of non-diabetic (white square), diabetic (black square), and SR1001 treated diabetic (grey circle) C57BL/6 mice. Each data point represents an individual retina from 4 different mice. Error bars represent the SEM, and *p*-value was first equated by two-way ANOVA analysis and then an unpaired *t*-test with Tukey’s post-hoc analysis, wherein * *p* < 0.01. All samples were collected 8 months after diabetic conditions were confirmed.

In the early stages of diabetic retinopathy and in this 8-month murine model, endothelial cells can die in the retinal capillaries, resulting in acellular and degenerative capillaries, which leads to capillary non-perfusion [6,35]. To determine if SR1001 is sufficient to halt capillary degeneration in the diabetic retina, we isolated the capillary beds of retinas (*n* = 4) from untreated non-diabetic and diabetic C57BL/6 mice, as well as SR1001 treated diabetic mice, 8 months after diabetes was confirmed. All acellular capillaries (representative examples are highlighted by red arrows in Figure 6A) were manually quantified. The number of acellular capillaries in the retinas of diabetic mice was significantly higher than in non-diabetic mice (Figure 6B). While the number of acellular capillaries in the retinas of diabetic mice receiving SR1001 injections was significantly decreased and similar to that of non-diabetic mice (Figure 6B). This indicates that SR1001 is sufficient to halt diabetes-mediated capillary non-perfusion, which is the hallmark of non-proliferative diabetic retinopathy. 

## 3. Discussion

Diabetic retinopathy is a diabetes-mediated retinal microvascular disease, and is one of the leading causes of vision loss within the working-age population worldwide [7,35]. Gradual, asymptomatic alterations in the retinal microvasculature can lead to capillary non-perfusion and vascular leakage, which are among the earliest detectable symptoms of non-proliferative diabetic retinopathy [11]. In response to this vasoregression, neovascularization in the retina is induced, leading to proliferative diabetic retinopathy [35]. The cause of diabetic retinopathy is multifactorial, but studies over the last decade provide strong evidence that diabetes induces low-grade inflammation, which leads to oxidative stress, enhanced VEGF production, and vascular permeability in the retina. Retinal inflammation then initiates capillary non-perfusion and degeneration [36,37,38,39]. This further enhances VEGF production and downstream signaling, which initiates neovascularization in the retina that leads to vision loss [7,11]. Although anti-VEGF treatments have helped a profuse number of diabetics, many are non-responders to this treatment [40,41,42,43]. It is still unknown why some diabetics respond to anti-VEGF treatments, while there is no therapeutic effect on others. Further, there is no therapeutics for non-proliferative diabetic retinopathy. Anti-VEGF treatments and ophthalmic surgeries are performed to treat diabetic macular edema and proliferative diabetic retinopathy. Hence, we still need to identify potential therapeutic targets that would inhibit the inflammatory-mediated progression of diabetic retinopathy, and enhance the efficacy of anti-VEGF treatments.

IL-17A has been identified as an important cytokine in the promotion of diabetes and the progression of diabetic complications [22,23,24,25,26]. IL-17A is not constitutively produced in healthy humans, but rather it is induced in inflammatory conditions. However, clinical diabetes studies have provided evidence that IL-17A is continuously produced in the blood of both Type I and Type II diabetics [20,21,22]. Further, levels of IL-17A have been correlated to the progression of multiple diabetic complications in Type I and Type II diabetic patients [19,29]. Recently, we established a pathological role for IL-17A in the onset of non-proliferative diabetic retinopathy in Streptozotocin (STZ)-induced diabetic mice [25,26]. We discovered that diabetes stimulates neutrophils and Th17 cells to produce IL-17A [25]. In diabetes, IL-17A-producing cells migrate through and adhere to the retinal vasculature, wherein IL-17A crosses the blood-retina-barrier and binds to its receptor, which is expressed on photoreceptors, Muller glia, and retinal endothelial cells [23,24,25]. IL-17A-dependent pathogenesis is then initiated through a cascade of signaling events downstream of the IL-17A receptor [23,26]. We further discovered that IL-17A signaling in retina cells induces VEGF production, vascular leakage, and capillary degeneration in diabetic mice [25,26]. Others have also demonstrated a role for IL-17A in retinal neural cell pathogenesis in murine models of diabetic retinopathy [23]. In these studies, it was determined that diabetic conditions induced Muller glia to produce IL-17A. In addition to stimulating IL-17A production, hyperglycemia also enhanced the expression of the IL-17A receptor in Muller glia. Muller glia was determined to enhance neuronal apoptosis, vascular leukostasis, and vascular permeability in the retina through an autocrine signaling cascade in Muller glia [23,24]. Taken together these previous studies provide evidence that diabetes-mediated IL-17A is pivotal in the onset of diabetic retinopathy.

Also, relevant is the significance of IL-17A in anti-VEGF drug resistance. Anti-VEGF drugs are currently used to treat different types of cancers (especially renal cancer), wet age-related macular degeneration, macular edema, and diabetic retinopathy [44,45,46,47,48]. In all of these diseases, the efficacy of anti-VEGF drugs in many patients has been limited by intrinsic or acquired drug resistance, for unknown reasons [49,50]. In cancer studies, it was determined that anti-VEGF resistance is driven by tumor-secreted factors, and that IL-17A was the most abundant protein secreted by tumor resistant cells [49,50,51]. Further studies showed that the use of anti-IL-17A significantly improved the antitumor activity of anti-VEGF treatments [50]. Although the mechanism has not yet been fully identified, it is known that IL-17A neutralization restores the efficacy of anti-angiogenic VEGF treatment in tumorigenesis [50]. Similarly, diabetes-mediated IL-17A enhances VEGF production, which probably leads to neovascularization seen in proliferative diabetic retinopathy. Hence, all therapeutic targets that could halt IL-17A production and IL-17-dependent retinal pathogenesis could be a potential therapeutic that could enhance the efficacy of anti-VEGF treatment, and delay the onset of diabetic retinopathy. Since diabetes-mediated IL-17A production is RORγt dependent, and there is a RORγt small molecule inhibitor (SR1001) that can pass the blood-retina-barrier, we postulated that RORγt would be a good therapeutic target and SR1001 would be a good drug candidate for the treatment of diabetic retinopathy. Accordingly, we examined the role of RORγt in the onset and progression of diabetic retinopathy, while also examining the potential of SR1001 as a therapeutic for diabetic retinopathy in this current study. 

The *RORC* gene transcribes RORγt, which is a protein that binds to DNA and act as a transcription factor in a ligand-dependent fashion [33,52]. RORγt is required for Th17 cell differentiation and IL-17A production [52]. Diabetes-mediated inflammation and hyperglycemia activate RORγt, wherein hyperglycemia-driven IL-6 and glucose sensitive stimulatory factors co-activate a STAT3 signaling cascade that activates RORγt [15,16]. Activation of RORγt then initiates nuclear translocation, where RORγt binds to the promoter region of the *Il17* gene, inducing IL-17A production [28]. Previous studies implicated that the RORγt/IL-17A axis played a pivotal role in the onset and progression of diabetic nephropathy [29,30]. Additionally, multiple IL-17A-producing cells that have been shown to play a role in the onset of diabetic retinopathy such as, microglia, neutrophils, and Th17 cells express RORγt [25,31,53]. In the current study, we provide evidence that the RORγt/IL-17A axis plays a pivotal role in the onset of diabetic retinopathy. Specifically, when RORγt was ablated in RORγt^−/−^ diabetic mice retinal inflammation was significantly decreased and oxidative stress was ameliorated. Both of these early stage retinal pathologies have been previously shown to lead to capillary non-perfusion and the onset of non-proliferative diabetic retinopathy [4,38]. Further, when T cells of diabetic wild type (C57BL/6) and RORγt^−/−^ mice were co-cultured with murine retinal endothelial cells (mREC), cell death was induced, however, mREC cell death was significantly decreased when mREC were cultured with T cells of RORγt^−/−^ diabetic mice. This suggests that RORγt plays a role in retinal endothelial cell death that leads to capillary non-perfusion and degeneration, which is the hallmark of clinical non-proliferative diabetic retinopathy. These studies demonstrate that diabetes-mediated RORγt can induce the onset of non-proliferative diabetic retinopathy, while previous studies determined that RORγt plays a role in the onset of diabetic nephropathy. Collectively this suggests that RORγt would be a good therapeutic target for multiple diabetic complications.

Structural studies of the ROR family identified a Liver X Receptor (LXR) agonist as a potent inverse agonist for RORγt, which led to the synthesis of SR1001 [54]. SR1001 is a ~480 Da small molecule ligand that increases the affinity of co-repressors while decreasing the affinity of co-activators when it binds to RORγt [34]. Further, SR1001 binding induces a conformational change in RORγt to prevent DNA binding and transcriptional activation [33,55]. Previously, SR1001 treatment has delayed the onset and progression of experimental autoimmune encephalomyelitis (EAE) and multiple sclerosis (MS) in murine models [30,55]. In non-obese diabetic (NOD) mice, SR1001 treatment prevented Th17 cell differentiation and IL-17A production, which halted Type I diabetes progression and insulitis [30]. Further in oxygen induced retinopathy (OIR), SR1001 treatment was found to limit neovascularization, vascular leakage, and the production of VEGF [31,32]. In our current study, we discovered that SR1001 treatment ablated diabetes-mediated IL-17A production, which in turn significantly decreased leukostasis in the retinal vasculature, retinal inflammation, and VEGF production. Finally, SR1001 treatment ameliorated retinal capillary degeneration, which is ratified as clinical non-proliferative diabetic retinopathy in this murine model. Taken together, these results suggest that RORγt small molecule inihibitor-SR1001 could be a potential therapeutic for diabetic retinopathy.

There is an FDA approved anti-IL-17A drug that is currently being administered to patients by intravenous injections for treatment of plaque psoriasis, psoriatic arthritis, and ankylosing spondylitis. However, anti-IL-17A is a humanized monoclonal anti-IL-17A antibody that will not pass the blood–retina-barrier. Intravitreal injections of anti-IL-17A are still a possible therapeutic for diabetic retinopathy that we are further investigating. However, because diabetes induces systemic IL-17A production that has been determined to play a pivotal role in the progression of multiple diabetic complications, it would be beneficial to identify a therapeutic that could halt systemic IL-17-dependent pathology and cross the blood–retina-barrier. We conclude that this study provides strong evidence that RORγt inhibitor-SR1001 would be a good therapeutic candidate for the onset and progression of diabetic retinopathy.

## 4. Materials and Methods

### 4.1. Streptozotocin (STZ)-Induced Diabetic Mice

CWRU IACUC and LSCVAMC ACORP approved the animal protocols employed in this study. C57BL/6, RORγt-GFP (heterozygous *Rorc*^+/GFP^: express functional RORγt that fluoresces green), and RORγt^−/−^ (homozygous *Rorc*^GFP/GFP^: do not express functional RORγt) mice were obtained from Jackson Laboratories. Diabetes was induced in 8–10-week-old male mice by intraperitoneal injections of (STZ) streptozotocin (60 mg/kg) on 5 consecutive days. Diabetes was defined by 6 h fasted blood glucose concentrations greater than 275 mg/dl, which was verified using glucose-dehydrogenase-based strips 17 days after the last STZ injection (Day 22). Hyperglycemia was quantified by hemoglobin A1c levels using the Crystal Chem Mouse A1c kit; 6 weeks and 29 weeks after diabetes was confirmed. Insulin (0-0.2 U) was administered as needed to maintain proper body weight. Retinal inflammation and leukostasis analyses were performed 2 months after diabetic conditions were confirmed, while capillary degeneration analyses were performed 8 months after diabetic conditions were confirmed. As previously described, these are the optimal time points for these analyses in this murine model [37,38,56]. 

### 4.2. Retinal Vasculature Staining and Leukostasis Analysis

Retinal vasculature was stained and leukostasis was analyzed as previously described [4,6]. Saline was perfused into the aorta to clear non-adherent leukocytes, then either 10 mL of Rhodamine (Figure 2) or Fluorescein (Figure 5) labeled Concanavalin A lectin (1 mg/mL in PBS; Vector laboratories) was perfused to stain vasculature. Retina flat mounts were imaged by fluorescent stereoscope, and the number of leukocytes adhered to the vasculature wall were counted.

### 4.3. Flow Cytometry of Retina Cells

Retinas were digested using the Papain Dissociation System (Worthington), incubated for 2 h at 37 °C in collagenase (80 U/mL; Sigma-Aldrich), and cells collected. Six retinas of three RORγt-GFP mice were pooled for flow cytometry analysis, whereas 5 of these pooled samples were analyzed (*n* = 15 mice/group). For apoptosis analysis, the Annexin V detection kit (eBioscience) was used according to manufacturer’s instructions. Cells were analyzed using a C6 Accuri flow cytometer (BD Bioscience); gates were set to isotype controls, and compensated using FlowJo software.

### 4.4. Murine Retinal Endothelial Cells

Murine retinal endothelial cells (mREC) originally were a kind gift of Dr. Nader Sheibani and isolated from the retinas of C57BL/6 mice as previously described [57]. Retinas were digested in collagenase and mREC were purified using magnetic beads coated with endothelial cell adhesion molecule-1, bound VE-Cadherin positive cells were isolated, and mREC purity was confirmed to be >99% pure.

### 4.5. Retinal Endothelial Cell Vviability Assays

Murine retinal endothelial cells (mREC) were cultured at 80% confluency, as previously described [26,36]. Spleens were removed, a single-cell suspension was generated, and incubated in erythrocyte lysis buffer (eBioscience) for 5 min at 37 °C, and then washed and counted. CD3^+^ T cells were negatively selected from isolated splenocytes using mouse CD3+ T cell enrichment columns (R&D). Cells were washed, and 1 × 10^5^ T cells of non-diabetic or diabetic C57BL/6 or RORγt^−/−^ mice were co-cultured with 3 × 10^5^ murine retinal endothelial cells per well. Cells were stained with 10 μg/mL Propidium Iodide, incubated for 48 h, and imaged using a Leica DMI 600B inverted microscope. Additionally, co-cultured cells were then stained with 0.25 μg/tube CD144 (BD Bioscience) for flow cytometry quantification of cell viability, as previously described [36]. Alternatively, co-cultured cells were incubated with Annexin V (eBioscience) antibodies for apoptosis analysis per manufacturer’s directions.

### 4.6. Quantification of Reactive Oxygen Species (ROS)

Blood vessels were perfused, retinas were isolated and incubated in Krebs-HEPES buffer (with 5 mmol/L glucose) for 25 min at 37 °C in 5% CO_2_. Luminescence was measured 5 min after the addition of 0.5 mmol/L lucigenin, as previously described [3].

### 4.7. ELISA Analysis

Sera or retina protein lysates were collected. Retina protein lysates were pooled from 3 retinas, and 3-pooled samples were analyzed using 2-site mIL-17A, mTNF-α, and mVEGF ELISA according to the manufacturer’s directions (R&D Biosciences).

### 4.8. RORγt Small Molecule Inhibitor-SR1001 Treatment Regimen

SR1001 is a small molecule inverse agonist of RORγt. SR1001 is ~480 Da, and represses transcriptional activity at the *Il17* promoter by inhibiting co-activator interaction and promoting the binding of co-repressors [34]. Lyophilized SR1001 was suspended in DMSO (5 mg/250 μL), and diluted in sterile saline to 5 μM/100 μL (final DMSO concentration = 0.125%). C57BL/6 diabetic mice received weekly subcutaneous injections, which started one week after diabetic conditions were confirmed (Day 29). Mice analyzed at the 2-month time point received 7 injections, while mice analyzed at the 8-month time point received 28 injections. Toxicity parameters for SR1001 were defined by weekly body weight measures, epidemiology of lethargy and mortality rate, and organ appearance by autopsy. No toxicity was observed in any of these SR1001 treatment regimens.

### 4.9. Capillary Degeneration in the Retina

Acellular capillaries were quantified in the retinal vasculature as previously described [6,7]. Eyes were fixed with 10% formalin. Retinas were incubated in elastase for 2 h followed by acidic buffer overnight. Retinal vasculature was mechanically isolated and stained with hematoxylin and periodic acid-Schiff. Acellular capillaries were quantified in 7 field areas between the optic nerve and the periphery (200× *g* magnification). Representative pictures were taken using a 40× objective mounted on an Olympus BX-60 microscope, using a Q-imaging Retiga Exi camera and Metamorph imaging software.

### 4.10. Statistical Analysis

Statistical analysis was performed using a two-way ANOVA and an unpaired t-test with Tukey’s post-hoc analysis (Prism, GraphPad Software). A *p*-value < 0.05 was considered significant. Error bars represent the standard error of the mean (SEM).
